# Effectiveness of a digi-physical tool and working method for paediatric obesity treatment in Abu Dhabi: a non-inferiority intervention study using an external historical comparator

**DOI:** 10.1186/s12887-026-07623-7

**Published:** 2026-09-10

**Authors:** Louise Lindberg, Reem Hassan Beck, Pernilla Danielsson, Timmy Nielsen, Asmahan Abdalla, Peter Ekstedt, Deepti Chaturvedi, Nabras Al Qahtani, Claude Marcus, Asma Deeb

**Affiliations:** 1 Division of Pediatrics, Department of Clinical Science, Intervention and Technology, Karolinska Institutet, Stockholm, Sweden; 2 Evira AB, Triewaldsgränd 2, Stockholm, Sweden; 3 Paediatric Endocrine Division, Sheikh Shakhbout Medical City, Abu Dhabi, United Arab Emirates; 4 Swedish Health, Dubai, United Arab Emirates; 5 Faculty of Health & Science, Khalifa University, Abu Dhabi, United Arab Emirates

**Keywords:** Paediatric obesity, Digital health, Treatment outcome, Home-weighing, Non-inferiority intervention study

## Abstract

**Background:**

Effective paediatric obesity treatment requires high intensity, scalable interventions. A digi-physical tool for paediatric obesity treatment has shown positive results in Stockholm, Sweden. This study evaluates whether the same treatment method is effective in a different cultural setting.

**Methods:**

This non-inferiority intervention study, using an external historical comparator, included 60 consecutively recruited children aged 6–15.9 years with obesity who initiated treatment at Sheikh Shakhbout Medical City in Abu Dhabi between June and December 2023. Patients were treated with Evira, a digi-physical tool and working method enabling high intensity individualized care, real-time monitoring, and interactive patient-clinician communication. The primary outcome was BMI z-score change at 26 weeks. Non-inferiority was assessed using a predefined margin of 0.10 BMI z-score, with outcomes compared to a prior published trial in Stockholm (*n* = 107).

**Results:**

A total of 112 children were included in the analysis (Abu Dhabi cohort, *n* = 35; Stockholm cohort, *n* = 77). The adjusted mean change in BMI z-score was − 0.20 (95% CI: − 0.28, − 0.12) in the Abu Dhabi cohort and − 0.20 (– 0.26, − 0.14) in the Stockholm cohort (*p* = 0.88). Non-inferiority was confirmed, (predefined margin 0.10 was not exceeded). A clinically significant BMI z-score reduction (≥ 0.20 units) was achieved by 45.7% of participants in Abu Dhabi and 36.4% in Stockholm (*p* = 0.35). Non-retention rates at 26 weeks were 41.7% vs. 28.0%, respectively (*p* = 0.07).

**Conclusions:**

The findings provide promising evidence that treatment outcomes achieved with the digi-physical treatment tool were comparable in the Abu Dhabi and Stockholm cohorts, supporting its feasibility in a second cultural and healthcare setting.

**Supplementary Information:**

The online version contains supplementary material available at https://doi.org/10.1186/s12887-026-07623-7.

## Introduction

Childhood obesity is a complex, multifaceted condition influenced by both genetic and environmental factors [1]. Globally, nearly 160 million children and adolescents are living with obesity, making it one of the most urgent public health challenges [2]. Children and adolescents with obesity face an increased risk for complications, including metabolic co-morbidities, mental health issues, and premature mortality [3–6].

Obesity affects approximately one in five children in the United Arab Emirates (UAE) [7], driven by a combination of sedentary lifestyles, high consumption of energy-dense fast food, and genetic predispositions [8]. Over the past two decades, these factors have contributed to a concerning increase in obesity prevalence, particularly among children aged 11 to 14 years [9].

Standard approaches to paediatric obesity treatment typically involve lifestyle modifications aimed at improving diet and increasing physical activity [10]. However, despite ongoing efforts, evidence indicates that traditional treatment methods yield suboptimal outcomes [11–14], with limited long-term success in achieving sustained weight reduction [15]. Recent advances, including pharmacological treatments for adolescents, have shown promising results [16–18], but face challenges such as financial and accessibility barriers.

Intensive behavioral programs involving frequent interactions, with at least 26 contact hours during the first year, have shown better outcomes [15]. However, these programs are challenging to implement due to resource constraints and the significant time commitments required from families and clinicians. Additionally, many communities lack access to such high-intensity programs [10].

Digital health interventions have emerged as promising solutions to address these challenges. Digital tools facilitate remote monitoring and communication between patients and healthcare providers, reducing the need for frequent in-person visits. These tools have shown positive reception from patients and families [19], but many rely on self-reported data, which may limit their precision and effectiveness [20].

The digital treatment tool integrated into clinical care–Evira–and its associated methodology (described in detail below) enables frequent communication while reducing the need for time-consuming in-person visits. Previous studies have shown promising results, including a 6-month feasibility study [19], a 12-month pragmatic trial [21], and a 3-year follow-up trial [22]. The pragmatic trial conducted in Stockholm, Sweden, demonstrated significant reductions in body mass index (BMI) z-score over one year [21], with patients maintaining their weight loss for up to three years [22], highlighting the tool’s long-term potential in paediatric obesity management. While the trial included a diverse patient population, its efficacy has not yet been evaluated in a different cultural context. The current study aimed to evaluate the effectiveness of the digital treatment tool in a new setting–the Sheikh Shakhbout Medical City (SSMC) in Abu Dhabi–over a 26-week period and to assess whether treatment outcomes were non-inferior to those observed in a previous pragmatic trial conducted in Stockholm.

## Methods

### Study population

This intervention study, using an external historical comparator, was conducted at the paediatric endocrine clinic SSMC, Abu Dhabi. Children aged 6 to 15.9 years with obesity, defined according to the International Obesity Task Force (IOTF) age- and sex-specific BMI cut-offs [23], who attended SSMC between June and December 2023 were eligible for inclusion. Families were required to be able to communicate in the local language and have access to a smartphone and an email address. Exclusion criteria were severe obesity (iso-BMI > 40 kg/m^2^), endocrine disorders other than well controlled hypothyroidism, treatment for eating disorders, depression and other psychiatric disorders during the last 6 months before inclusion or observed at the inclusion screening, pharmacological treatment affecting weight control, hypothalamic or monogenic obesity, or severe neuropsychiatric disorders.

**Fig. 1:**
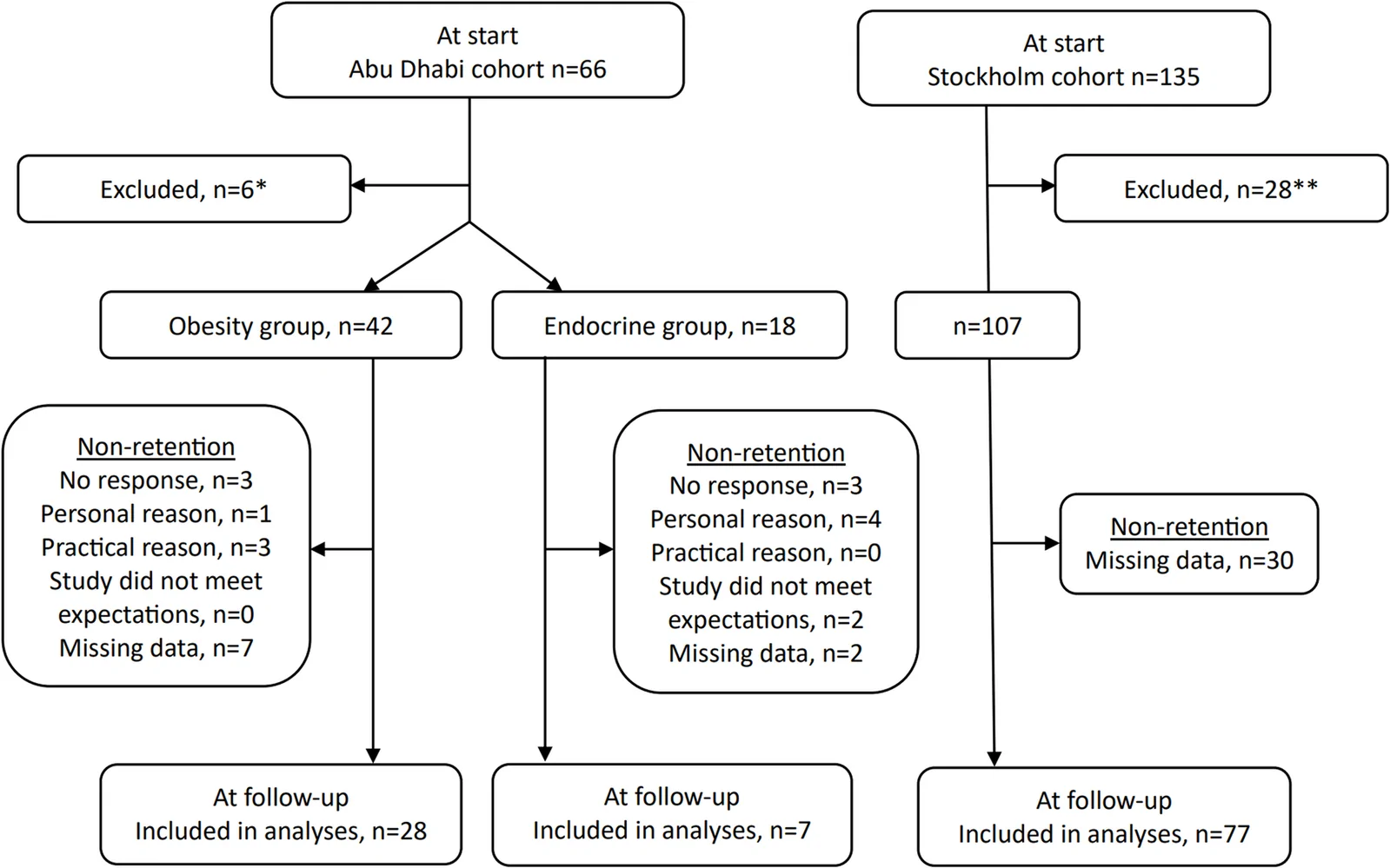
Flowchart of participants * iso-BMI < 30 *n* = 2, iso-BMI > 40 *n* = 4 ** Overweight *n* = 26, declined the support system *n* = 2

Participants in the Abu Dhabi cohort were further divided into two subgroups based on their primary reason for referral: (1) obesity and (2) general endocrine concerns. The latter group primarily attended the clinic for conditions such as short stature or thyroid concern but were also identified as having obesity and were therefore offered participation in the study. A flowchart of participant inclusion and follow-up is illustrated in Fig. 1.

The study received ethical approval from the Institution Review Board of SSMC, Abu Dhabi (SSMCREC-395) as well as the Stockholm Ethics Committee (approval no. 2018/1413–31, amendment 2024-06085-02), and was performed in accordance with the Helsinki Declaration. The study is registered on Clinicaltrials.gov (ID: NCT06665893).

### The digi-physical tool and working method

**Fig. 2:**
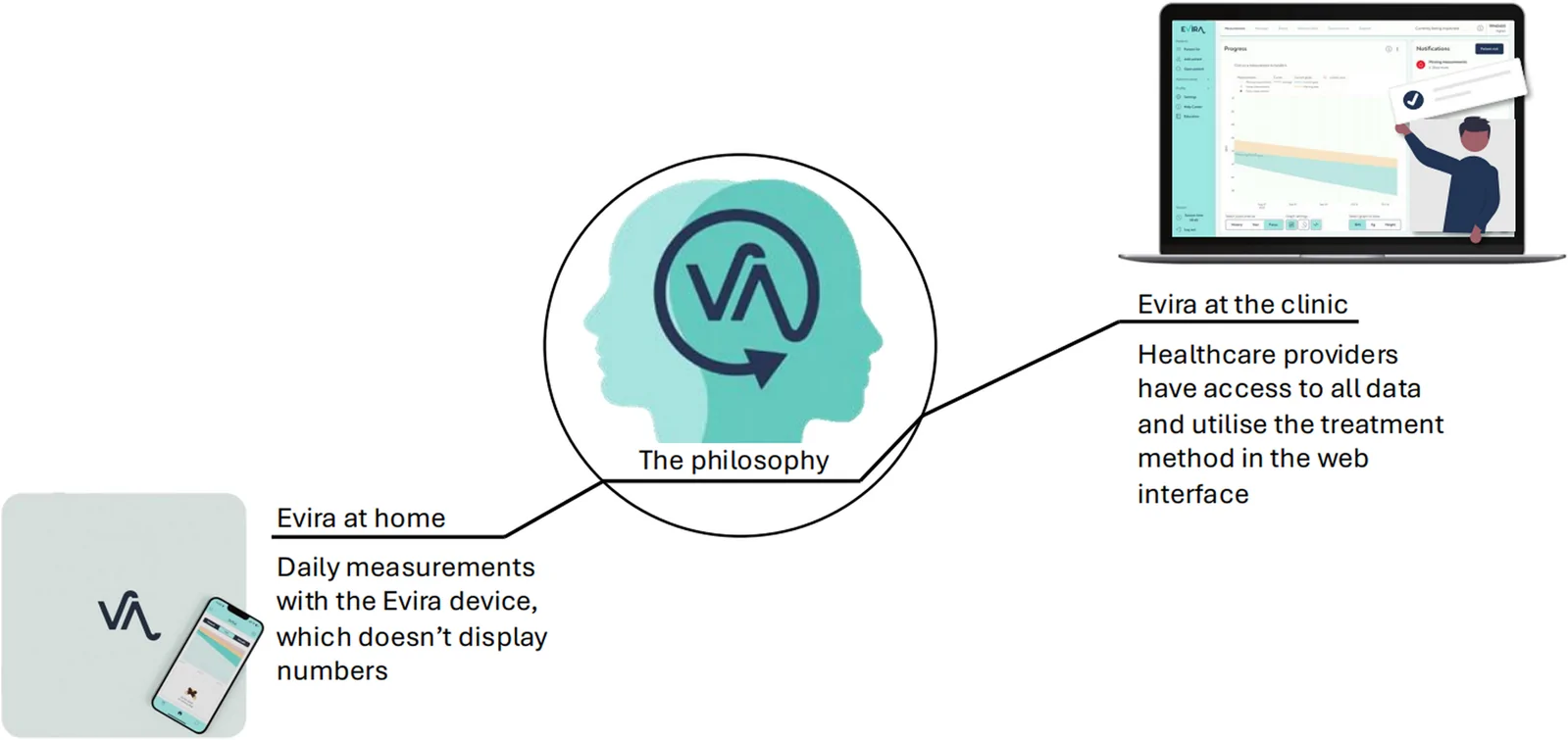
The digi-physical treatment tool

The Evira digital tool incorporates a digitless body scale, with results transmitted to a mobile application on the parents’ phone and accessible to healthcare providers via a web-based clinic interface for efficient, high-intensity, patient management (Fig. 2). The treatment approach emphasizes individualized outcome-based goals, collaboratively set by clinical staff together with the families. Patients are encouraged to conduct daily home measurements, with results displayed graphically in the app as relative progress over time, based on BMI z-score. Instead of prescribing specific dietary or physical activity advice, the accompanying working method centers on family empowerment, encouraging self-reflection and supporting families in devising their own strategies to achieve weight goals. The tool also enables fast and easy communication between the family and the clinic. Clinical staff provide support, address barriers, and engage with families if weight targets are unmet or daily measurements are missed. This approach fosters a shared understanding of treatment progress and enables clinical staff to provide timely support based on real-time data.

### Study procedure

Eligible patients were invited to participate after receiving oral and written information about the study from one of the study clinicians. Informed consent was obtained from the parents or legal guardians, with voluntary participation and the option to withdraw at any time without impacting their ongoing care. Six patients in the Abu Dhabi cohort had an iso-BMI below 30 or above 40 at baseline and were therefore excluded from the analyses (Fig. 1). Study visits were scheduled at baseline, 3 months and 6 months, during which weight and height measurements were recorded. At each visit, the clinical staff, in collaboration with the families, set individualized weight loss targets for the upcoming three months, which is visualized in both the web-interface and the app. At baseline and 6 months, additional clinical examinations, including anthropometric measures and blood sampling (e.g. insulin, fasting glucose, HbA1C, liver and cholesterol markers) were conducted.

Families were provided with the digital measuring device and assisted with installing the mobile app on the parents’ phone, and where applicable, the child’s phone during the baseline visit or a separate enrollment visit. Study data–including family background information, clinical measures, number of physical visits, cancellations and missed visits, side effects, and responses to questionnaires on eating disorders, quality of life, and treatment satisfaction–were documented in an electronic Case Report Form (e-CRF).

The study team, consisting of a research clinical scientist (RHB) and paediatric endocrinologists (AD, DC, NAQ), were responsible for patient enrollment, digital support, and follow-up. The clinical staff trained families on using the measuring device and app and emphasized the importance of regular communication between visits. Clinical staff were instructed to refrain from providing specific advice, instead encouraging families to take ownership of the treatment process by developing their own tailored strategies. To support a consistent approach, an accompanying treatment manual was available to the clinical staff. The manual included guidance on the Evira philosophy, including empowerment principles and communication strategies. During in-person visits, clinicians could address more sensitive issues, such as familial conflicts around mealtime or challenging food situations. Training and oversight were provided by collaborators from Sweden.

### The Stockholm cohort

The digi-physical treatment tool was previously evaluated by Hagman and colleagues in a pragmatic clinical trial involving 107 patients who began treatment between August 2018 and March 2019 at an obesity clinic in Stockholm, Sweden [21]. The study procedure was the same as that used at SSMC. The objective in the present study was not to demonstrate superiority, but to assess whether outcomes achieved with an established digi-physical treatment tool could be maintained when implemented in a different healthcare and cultural context. Therefore, a non-inferiority design using the Stockholm cohort from the previous pragmatic trial as a historical comparator was considered appropriate. Patients in the Stockholm cohort were followed for one year. To ensure comparable follow-up durations, outcomes from the Abu Dhabi cohort were compared with the 6-month data from the Stockholm cohort previously described by Hagman and colleagues [21].

### Outcome measures

The primary outcome was the change in BMI z-score from baseline to 26 weeks, defined according to the IOTF criteria [23]. A 26-week follow-up period was selected as the primary endpoint to provide an initial proof-of-concept assessment of whether treatment outcomes achieved with the digi-physical treatment tool could be replicated in a new cultural and healthcare setting. The study hypothesized that the mean BMI z-score change in the Abu Dhabi cohort would be non-inferior to that in the Stockholm cohort, using a predefined non-inferiority margin of 0.10 BMI z-score units. The margin represents the maximum acceptable difference while still supporting comparable treatment effectiveness between the cohorts. Follow-up was conducted at 26 weeks +/- 14 days (166–194 days).

Secondary outcomes included the proportion of children achieving a clinically significant reduction in BMI z-score (defined as a decrease of 0.20 BMI z-score units or more) [24], the proportion of children in obesity remission, non-retention rate, and treatment adherence. Treatment adherence was assessed through measurement frequency and engagement in digital communication, as daily home measurements and patient-clinic communication constitute key components of the digi-physical treatment tool. Measurement frequency was defined as the mean number of home measurements per week during month 1 (days 1–28) and month 6 (days 150–177), and engagement in digital communication as the mean number of messages sent from (a) the clinic to the patient and (b) the patient to the clinic over the entire 26-week treatment period (days 1–194).

Degree of obesity was categorized as “obesity Class I” or “obesity Class II” according to Cole et al. [23].

### Statistical analysis

Descriptive statistics are presented as mean (min–max) for continuous variables and as frequencies and percentages for categorical variables. For continuous variables, an independent t-test was used for normally distributed data, while the Wilcoxon Rank-Sum test was applied for non-normally distributed data. For categorical variables, chi-square tests were used to assess group differences.

Change in BMI z-score from baseline to 26 weeks was calculated for each cohort and within subgroups. A generalized linear model (PROC GENMOD) was used to assess change in BMI z-score, adjusted for sex, age- and degree of obesity at treatment initiation. The adjusted BMI z-score change and corresponding 95% confidence interval (CI) were estimated for each cohort and subgroup.

To evaluate non-inferiority, an unadjusted generalized linear model (PROC GENMOD) with gamma distribution and log-link function was applied. This approach was chosen because the distribution of BMI z-score change deviated from normality. Non-inferiority was confirmed if the upper bound of the 95% CI did not exceed the predefined margin of 0.10 BMI z-score units.

Differences in non-retention rates between cohorts were assessed using Chi-square test. Univariate logistic regression was used to estimate odds ratios (ORs) and 95% CIs for non-retention. Treatment adherence is presented as mean and standard deviation (SD). For all analyses, only observed data was used. A p value < 0.05 was considered statistically significant.

The sample size was calculated to account for an expected drop-out rate of 50%, based on previous obesity treatment studies [25]. A minimum of 30 participants with follow-up data was considered sufficient to evaluate the intervention in a new cultural setting and compare treatment outcomes with those observed in the Stockholm cohort.

SAS Statistical software (version 9.4, SAS Institute Inc., Cary, NC) was used for the analyses.

## Results

### Study population

Of 66 consecutively recruited children and adolescents in the Abu Dhabi cohort, 60 (91%) were included after inclusion and exclusion criteria were applied (47% males; 95% born in the UAE; Obesity group *n* = 42; Endocrine group, *n* = 18). At treatment initiation, mean (min-max) age was 11.7 (6.7–15.7) years and mean (min-max) BMI z-score was 2.95 (2.28–3.75) units in the Abu Dhabi cohort (Table 1). In the Stockholm cohort, 107 of 135 (79%; 67% males) patients were included, with a mean (min–max) age of 11.9 (4.1–17.4) years and a mean (min–max) BMI z-score of 2.81 (2.19–4.24) units at treatment initiation. The groups were similar in age (*p* = 0.57). Baseline characteristics are summarized in Table 1. At the 26-week follow-up, 35 patients (58.3%) in the Abu Dhabi cohort and 77 (72.0%) patients in the Stockholm cohort had eligible follow-up data (*p* = 0.07). The corresponding numbers in the subgroups were 28 (66.7%) patients in the Obesity group and 7 (38.9%) in the Endocrine group.

**Table 1:** Baseline characteristics of patients

	Abu Dhabi cohort		Stockholm cohort		
	Obesity group	General endocrine group	Total		p-value*
n (%)	42 (70)	18 (30)	60 (100)	107 (100)	
Males, n (%)	20 (47.6)	8 (44.4)	28 (46.7)	72 (67.3)	< 0.01
Age, mean (min–max)	11.7 (6.7–15.7)	11.5 (8.0–15.5)	11.7 (6.7–15.7)	11.9 (4.1–17.4)	0.57
BMI z-score, mean (min–max)	2.96 (2.28–3.75)	2.92 (2.30–3.68)	2.95 (2.28–3.75)	2.81 (2.19–4.24)	0.008
Weight status, n (%)					
Obesity class I	15 (35.7)	8 (44.4)	23 (38.3)	69 (64.5)	< 0.01
Obesity class II	27 (64.3)	10 (55.6)	37 (61.7)	38 (35.5)	< 0.01

*p-values between the Abu Dhabi cohort and the Stockholm cohort

### Change in BMI z-score

**Fig. 3:**
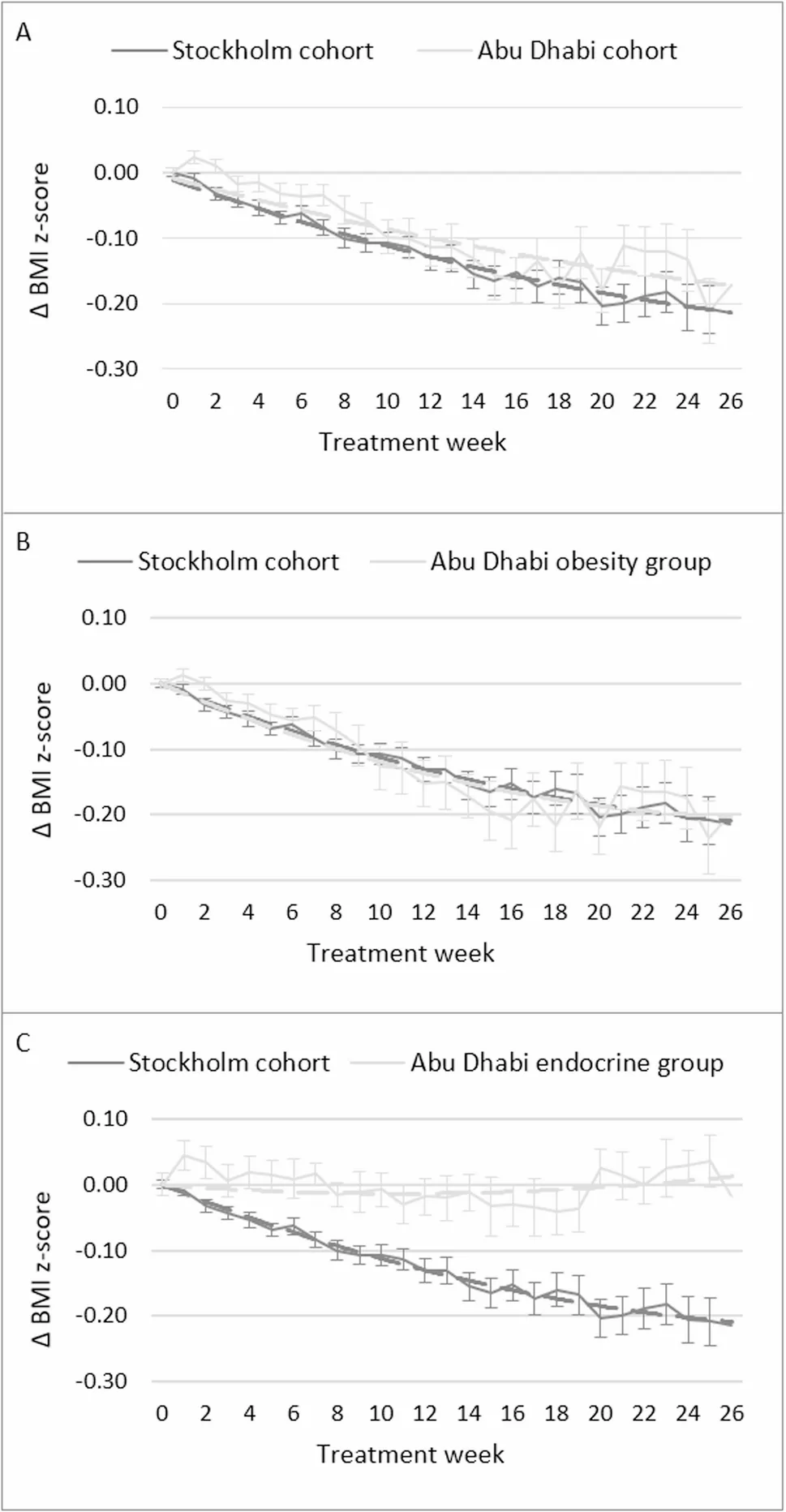
**a-c** Observed weekly mean change in BMI z-score from baseline to 26-weeks of treatment. Panel A compares the overall Abu Dhabi cohort with the Stockholm cohort. Panel B compares participants referred specifically for obesity treatment in Abu Dhabi with the Stockholm cohort. Panel C compares participants referred primarily for other endocrine concerns who were also identified as having obesity with the Stockholm cohort. Panel C is presented to illustrate the potential influence of referral reason, treatment motivation, and engagement on outcomes, and should not be interpreted as a direct comparison of intervention efficacy. The dark grey and light grey lines represent the Stockholm cohort and Abu Dhabi cohort/subgroups, respectively. Error bars present the standard error

Figure 3a-c illustrates the unadjusted continuous weekly mean change in BMI z-score from baseline to 26 weeks, including subgroup analyses based on referral reason. There was no significant difference in BMI z-score reduction between the cohorts. The adjusted mean (95% CI) change in BMI z-score was -0.20 (-0.28 to -0.12) for the Abu Dhabi cohort and -0.20 (-0.26 to -0.14) for the Stockholm cohort (*p* = 0.88; Table 2). Female sex was associated with a 0.10 greater decrease in BMI z-score (*p* = 0.04), while age and degree of obesity at treatment initiation were not significantly associated with the outcome. The change in BMI z-score across subgroups in the Abu Dhabi cohort is presented in Table 2. The unadjusted mean difference in BMI z-score change between the Abu Dhabi and Stockholm cohorts was 0.0004 (95% CI -0.004 to 0.005). The upper bound of the 95% CI (0.005) was below the predefined non-inferiority margin of 0.10, confirming non-inferiority.

**Table 2:** Treatment effect and adherence

	Abu Dhabi cohort		Stockholm cohort		
	Obesity group	General endocrine group	Total		p-value*
Adjusted mean change in BMI z-score (95% CI)**	-0.25 (-0.34 to -0.15)	-0.009 (-0.19 to 0.17)	-0.20 (-0.28 to -0.12)	-0.20 (-0.26 to -0.14)	0.88
Weekly measurement frequency, mean (SD)					
Month 1	5.2 (1.3)	4.3 (1.5)	4.9 (1.4)	5.0 (1.7)	0.18
Month 6	3.0 (1.8)	2.6 (1.5)	2.9 (1.7)	3.0 (1.9)	0.54
Messages over 26 weeks, mean (SD)					
From clinic to patient	32.3 (11.3)	28.6 (12.0)	31.2 (11.5)	29.9 (11.6)	0.50
From patient to clinic	16.3 (13.0)	12.5 (8.8)	15.5 (12.0)	16.7 (14.1)	0.49

*CI *confidence interval, *SD* standard deviation

*p-values between the Abu Dhabi cohort and the Stockholm cohort

**Adjusted for sex, age- and degree of obesity at treatment initiation

**Fig. 4:**
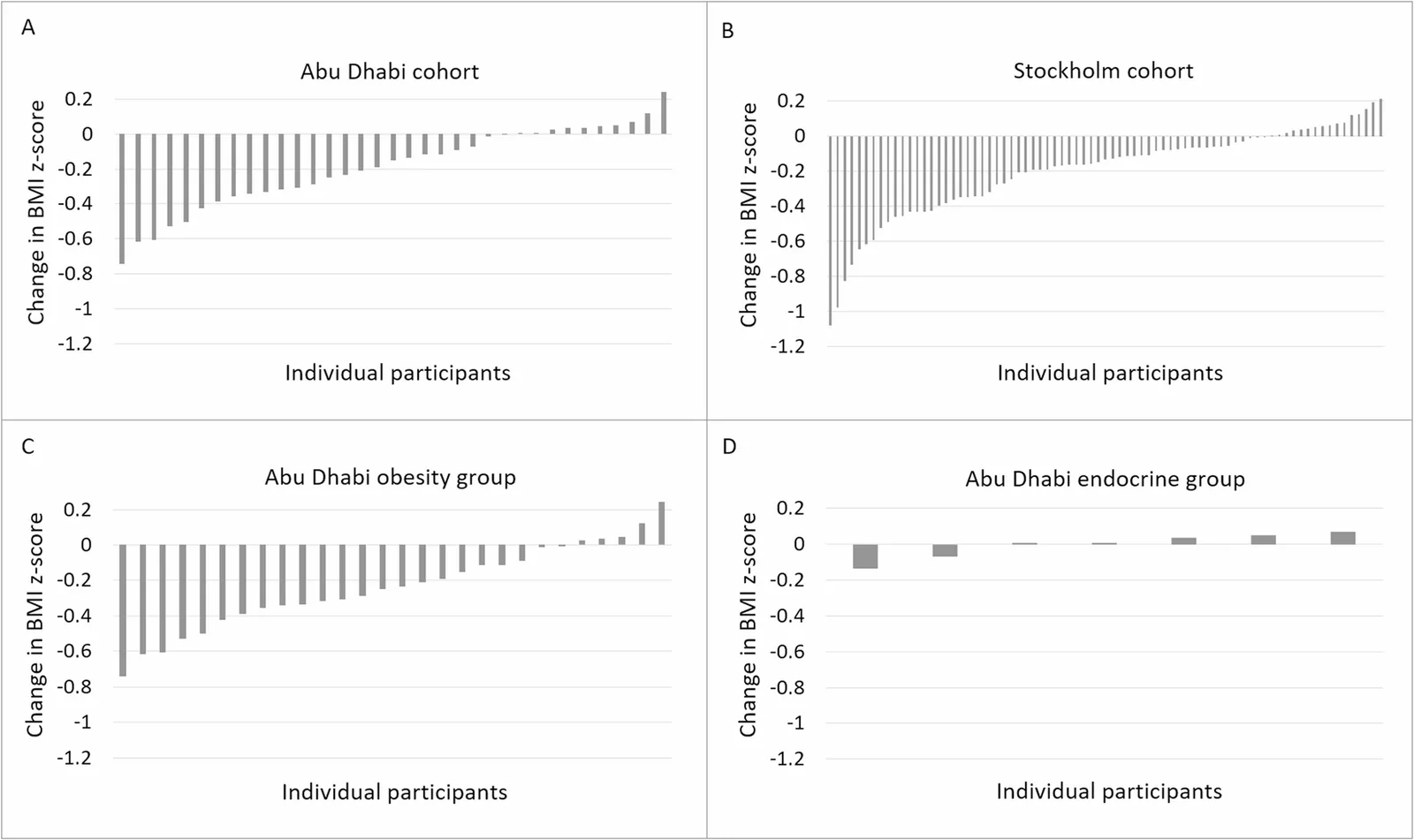
**a-d** Change in BMI z-score for each individual patient. Each bar represents one participant and indicates the change from the first to the last registered measurement. Participants are ordered according to the magnitude of BMI z-score change

Figure 4a-d shows the individual changes in BMI z-score for each patient from baseline to 26-weeks follow-up. Both cohorts exhibit a similar trend, with most patients achieving a decrease in BMI z-score during the treatment period. In the subgroups, the Abu Dhabi Endocrine group displays a more variable pattern.

The proportion of patients who achieved a clinically significant change in relative weight–defined as a decrease of 0.20 BMI z-score units or more after 26 weeks–was 45.7% in the Abu Dhabi cohort and 36.4% in the Stockholm cohort (*p* = 0.35). Among patients in the Abu Dhabi cohort, 14.3% went into obesity remission compared to 23.4% in the Stockholm cohort (*p* = 0.27). All patients in the Abu Dhabi cohort who achieved a clinically significant change in relative weight or obesity remission belonged to the Obesity group, whereas none belonged to the Endocrine group.

### Non-retention

At the 26-week follow-up, non-retention rates were 41.7% in the Abu Dhabi cohort and 28.0% in the Stockholm cohort (*p* = 0.07). Although non-retention was numerically higher in the Abu Dhabi cohort, univariate logistic regression analysis indicated no statistically significant difference between the cohorts (OR 1.83, 95% CI 0.94–3.57, *p* = 0.07). Reasons for non-retention are presented in Fig. 1. Children with 26-week follow-up data did not differ significantly from those without follow-up data with respect to sex, age- or BMI z-score at treatment initiation [see Additional file 1]. No adverse events were reported in the e-CRF during the follow-up.

### Measurement frequency and number of messages

The mean (SD) home weighing frequency was highest at the beginning of treatment, with approximately 4.9 (1.4) and 5.0 (1.7) measurements per patient per week during the first month in the Abu Dhabi and Stockholm cohorts, respectively (*p* = 0.18; Table 2). By the sixth month, the frequency declined to 2.9 (1.7) measurements per patient per week in the Abu Dhabi cohort and 3.0 (1.9) in the Stockholm cohort (*p* = 0.54; Table 2).

The mean (SD) number of messages sent from the clinic to the patient during the 26-week follow-up was 31.2 (11.5) in the Abu Dhabi cohort and 29.9 (11.6) in the Stockholm cohort (*p* = 0.50; Table 2). The mean (SD) number of messages sent from the patient to the clinic during the same period was 15.5 (12.0) in the Abu Dhabi cohort and 16.7 (14.1) in the Stockholm cohort (*p* = 0.49; Table 2). The corresponding values for measurement frequency and number of messages sent for the Abu Dhabi subgroups are provided in Table 2.

## Discussion

### Summary of findings

This 26-week intervention study evaluated the efficacy of a digi-physical treatment tool for paediatric obesity in Abu Dhabi and compared the outcomes to a similar pragmatic trial conducted in Stockholm, Sweden, using the same treatment tool [21]. The present study used a non-inferiority design and provides promising evidence that treatment outcomes in the Abu Dhabi cohort were comparable to those in the Stockholm cohort, supporting the tool’s feasibility and potential effectiveness in a new cultural setting. The upper confidence limit for the between-cohort difference (0.005 BMI z-score units) was substantially below the predefined non-inferiority margin of 0.10 units, suggesting that differences in treatment effect between the cohorts were unlikely to be clinically meaningful.

The Abu Dhabi cohort demonstrated a reduction in BMI z-score of -0.20 units, comparable to the results observed in the Stockholm cohort. This is a clinically significant reduction as it previously has been observed to be associated with a general reduction in risk markers [24]. Furthermore, recent evidence suggests that even short-term weight loss of this magnitude is linked to a substantial long-term reduction in the risk of developing type 2 diabetes [4]. Additionally, beneficial treatment responses in childhood have been shown to lower future morbidity and mortality risk in young adulthood [26]. A lower proportion remained in treatment at the 26-week follow-up in the Abu Dhabi cohort compared to the Stockholm cohort (58% vs. 72%), although not statistically significant. No adverse events were reported in the electronic case report form in the intervention study.

### Cultural and clinical context

The intervention in Abu Dhabi was implemented in a cohort divided into two groups based on the primary reason for their clinic visit. One group, consisting of patients referred specifically for obesity treatment, achieved weight loss outcomes comparable to those in Stockholm. Notably, this group showed no weight regain at the group level, which contrasts with the common pattern of weight regain following lifestyle interventions typically occurring within 3–4 months, even after successful initial weight loss [27, 28]. The findings from the present study are consistent with previous research suggesting that digital tools, such as Evira–combining daily home measuring with clinical digital supervision and support–can mitigate weight regain and support sustainable treatment outcomes across different cultural settings [21].

The second group, consisting of patients with obesity but who primarily were referred for other endocrine concerns (e.g., short stature, suspected hypothyroidism, puberty evaluations), demonstrated suboptimal weight outcomes. In this subgroup, adherence was somewhat lower, with less frequent home measurements and communication with clinical staff. One possible explanation is that families may have had a greater focus on the underlying endocrine condition rather than obesity, which may have influenced their engagement with the obesity treatment. Differences in patient and family motivation may also have contributed, as has been highlighted in previous research [29]. Families need time to discuss the challenges of obesity and deliberate on lifestyle modifications before initiating treatment. Successful lifestyle interventions for obesity require significant commitment from families, as they are implemented in the home environment and demand active engagement. The clinical staff’s role is to provide support and information, but meaningful weight loss becomes challenging to achieve without strong family motivation and active engagement.

### Challenges in retention

Non-retention is a well-documented challenge in paediatric obesity lifestyle interventions, including those incorporating mobile health technologies [30–32]. Non-retention rates vary depending on the type and duration of the intervention, typically ranging from 27% to 73% after 6 to 12 months of treatment [25]. Previous studies have demonstrated lower non-retention rates with the same digi-physical treatment tool compared with conventional treatment [19, 21, 22]. The observed non-retention in this study was lower than estimated at study initiation. Nevertheless, non-retention tended to be higher in the Abu Dhabi cohort than in the Stockholm cohort (42% vs. 28%, *p* = 0.07). Although this difference did not reach statistical significance, it may be clinically relevant and should be explored further in larger studies. Addressing non-retention may require greater emphasis on motivational work before treatment initiation to ensure families are fully prepared and committed. Improving treatment adherence is crucial, as unsuccessful treatment attempts or dropping out of treatment can be costly for providers and may increase the risk of anxiety and/or depression among patients [5], highlighting the importance of appropriate timing and readiness for treatment.

### Strengths and limitations

The study has several limitations that warrant consideration. First, the comparison relied on a historical Stockholm cohort rather than a contemporaneously recruited control group. As the digi-physical treatment tool and associated clinical workflows have continued to evolve over time, differences between study periods may have influenced the observed outcomes. The study was designed to assess whether treatment outcomes achieved with the digi-physical tool could be replicated in a different cultural and healthcare setting, rather than comparing the digital tool with standard care. The findings should therefore be interpreted as supporting the feasibility and potential effectiveness of the intervention in a new cultural setting.

The relatively short follow-up period of 26 weeks limits the assessment of long-term outcomes. In addition, the number of children included was relatively small, reducing statistical precision and limiting the ability to detect moderate differences between groups. This limitation is particularly relevant when interpreting findings from the subgroup analyses, especially those relating to the Endocrine group. Furthermore, the requirement for language proficiency and access to a smartphone and email may have introduced selection bias and limited generalizability by excluding families with lower digital literacy or reduced access to digital resources. In addition, both study settings were located in high-income countries with well-developed digital infrastructure, and caution is therefore warranted when generalizing the findings to other healthcare systems and populations.

The relatively high non-retention rate, particularly in the Abu Dhabi cohort, represents an important limitation of the present study. Although high attrition is common in real-world clinical practice, it also represents an important potential source of bias. Because the analyses were based on observed data only, treatment effects may have been overestimated if participants lost to follow-up experienced less favorable outcomes than those who remained in the study. Furthermore, as analyses were restricted to measurements obtained within a relatively narrow predefined follow-up window, some participants classified as having missing follow-up data may still have been engaged in treatment but lacked an eligible measurement within the specified time frame.

Despite these limitations, the study has several important strengths. Patients were consecutively recruited from routine clinical practice, increasing the generalizability of the findings. Frequent, objectively measured weight data were collected through the digital tool, reducing reliance on self-reported outcomes and enabling detailed longitudinal assessment of treatment response. In addition, the study represents the first evaluation of the digital treatment tool in a Middle Eastern setting and provides valuable data on its implementation and potential applicability across different cultural, clinical and healthcare settings.

## Conclusions

This 26-week intervention study demonstrates non-inferiority of a digi-physical treatment tool–combining high-intensity digital support and real-time family monitoring of treatment outcomes–for paediatric obesity treatment in Abu Dhabi compared to a similar cohort in Stockholm. The findings suggest promising evidence of the tool’s effectiveness in a second, distinct cultural and clinical setting. In addition, treatment success may be influenced by patient and family pre-treatment readiness and focus. Future studies evaluating the intervention across different socioeconomic and age groups, and in combination with pharmacological treatment, may further clarify its applicability in diverse patient populations.

## Supplementary Information

Supplementary Material 1.

## Data Availability

The data that support the findings of this study are available from Evira AB but restrictions apply to the availability of these data, which were used under license for the current study, and so are not publicly available. Data are however available from the authors upon reasonable request and with permission of Evira AB.

## Abbreviations

UAE: United Arab Emirates
BMI: Body mass index
SSMC: Sheikh Shakhbout Medical City
IOTF: International Obesity Task Force
e-CRF: Electronic Case Report Form
CI: Confidence interval
OR: Odds ratio
SD: Standard deviation

## References

[1] Marcus C, Danielsson P, Hagman E. Pediatric obesity—Long-term consequences and effect of weight loss. J Intern Med. 2022;292(6):870–91. 10.1111/joim.13547. 35883220 PMC9805112

[2] Phelps NH, Singleton RK, Zhou B, Heap RA, Mishra A, Bennett JE, et al. Worldwide trends in underweight and obesity from 1990 to 2022: a pooled analysis of 3663 population-representative studies with 222 million children, adolescents, and adults. Lancet. 2024;403(10431):1027–50. 10.1016/S0140-6736(23)02750-2. 38432237 PMC7615769

[3] Hagman E, Danielsson P, Elimam A, Marcus C. The effect of weight loss and weight gain on blood pressure in children and adolescents with obesity. Int J Obes. 2019;43(10):1988–94. 10.1038/s41366-019-0384-2. 31152153

[4] Putri RR, Casswall T, Danielsson P, Marcus C, Hagman E. Steatotic Liver Disease in Pediatric Obesity and Increased Risk for Youth-Onset Type 2 Diabetes. Diabetes Care. 2024;dc241236. 10.2337/dc24-1236. PMC11655408 39373987

[5] Lindberg L, Hagman E, Danielsson P, Marcus C, Persson M. Anxiety and depression in children and adolescents with obesity: A nationwide study in Sweden. BMC Med. 2020;18(1). 10.1186/s12916-020-1498-z. PMC7033939 32079538

[6] Lindberg L, Danielsson P, Persson M, Marcus C, Hagman E. Association of childhood obesity with risk of early all-cause and cause-specific mortality: A swedish prospective cohort study. PLoS Med. 2020;17(3). 10.1371/JOURNAL.PMED.1003078. PMC7080224 32187177

[7] Baniissa W, Radwan H, Rossiter R, Fakhry R, Al-Yateem N, Al-Shujairi A, et al. Prevalence and determinants of overweight/obesity among school-aged adolescents in the United Arab Emirates: a cross-sectional study of private and public schools. BMJ Open. 2020;10(12):e038667. 10.1136/bmjopen-2020-038667. 33310793 PMC7735131

[8] Al Sabbah H, Assaf EA, Al-Jawaldeh A, AlSammach AS, Madi H, Khamis Al Ali N, et al. Nutrition Situation Analysis in the UAE: A Review Study. Nutrients. 2023;15(2). 10.3390/nu15020363. PMC9861891 36678240

[9] AlBlooshi A, Shaban S, AlTunaiji M, Fares N, AlShehhi L, AlShehhi H, et al. Increasing obesity rates in school children in United Arab Emirates. Obes Sci Pract. 2016;2(2):196–202. 10.1002/osp4.37. 27818779 PMC5074293

[10] Hampl SE, Hassink SG, Skinner AC, Armstrong SC, Barlow SE, Bolling CF, et al. Clinical Practice Guideline for the Evaluation and Treatment of Children and Adolescents With Obesity. Pediatrics. 2023;151(2):e2022060640. 10.1542/peds.2022-060640. 36622115

[11] Hagman E, Danielsson P, Lindberg L, Marcus C. Paediatric obesity treatment during 14 years in Sweden: Lessons from the Swedish Childhood Obesity Treatment Register—BORIS. Pediatr Obes. 2020;15(7). 10.1111/ijpo.12626. 32074662

[12] Ek A, Lewis Chamberlain K, Sorjonen K, Hammar U, Etminan Malek M, Sandvik P, et al. A Parent Treatment Program for Preschoolers With Obesity: A Randomized Controlled Trial. Pediatrics. 2019;144(2). 10.1542/peds.2018-3457. PMC8853645 31300528

[13] Mead E, Brown T, Rees K, Azevedo LB, Whittaker V, Jones D, et al. Diet, physical activity and behavioural interventions for the treatment of overweight or obese children from the age of 6 to 11 years. Cochrane Database Syst Rev. 2017;6(6):CD012651. 10.1002/14651858.CD012651. 28639319 PMC6481885

[14] Al-Khudairy L, Loveman E, Colquitt JL, Mead E, Johnson RE, Fraser H, et al. Diet, physical activity and behavioural interventions for the treatment of overweight or obese adolescents aged 12 to 17 years. Cochrane Database Syst Rev. 2017;6(6):CD012691. 10.1002/14651858. 28639320 PMC6481371

[15] O’Connor EA, Evans CV, Henninger M, Redmond N, Senger CA. Interventions for Weight Management in Children and Adolescents: Updated Evidence Report and Systematic Review for the US Preventive Services Task Force. JAMA. 2024;332(3):233–48. 10.1001/jama.2024.6739.38888913

[16] Kelly AS, Auerbach P, Barrientos-Perez M, Gies I, Hale PM, Marcus C, et al. A Randomized, Controlled Trial of Liraglutide for Adolescents with Obesity. N Engl J Med. 2020;382(22):2117–28. 10.1056/nejmoa1916038. 32233338

[17] Kim A, Nguyen J, Babaei M, Kim A, Geller DH, Vidmar AP. A Narrative Review: Phentermine and Topiramate for the Treatment of Pediatric Obesity. Adolesc Health Med Ther. 2023;14:125–40. 10.2147/AHMT.S383454. 37641650 PMC10460571

[18] Weghuber D, Barrett T, Barrientos-Pérez M, Gies I, Hesse D, Jeppesen OK, et al. Once-Weekly Semaglutide in Adolescents with Obesity. N Engl J Med. 2022;387(24):2245–57. 10.1056/nejmoa2208601. 36322838 PMC9997064

[19] Johansson L, Hagman E, Danielsson P. A novel interactive mobile health support system for pediatric obesity treatment: A randomized controlled feasibility trial. BMC Pediatr. 2020;20(1). 10.1186/s12887-020-02338-9. PMC7513491 32967638

[20] Lei S, Inojosa JRM, Kumar S, Lee AT, Scott CG, Lerman A, et al. Effectiveness of a Weight Loss Program Using Digital Health in Adolescents and Preadolescents. Child Obes. 2021;17(5):311–21. 10.1089/chi.2020.0317. 33826417 PMC8236388

[21] Hagman E, Johansson L, Kollin C, Marcus E, Drangel A, Marcus L, et al. Effect of an interactive mobile health support system and daily weight measurements for pediatric obesity treatment, a 1-year pragmatical clinical trial. Int J Obes. 2022 Aug;1. 10.1038/s41366-022-01146-8. PMC9314258 35641569

[22] Hagman E, Lindberg L, Putri RR, Drangel A, Marcus C, Danielsson P. Long-term results of a digital treatment tool as an add-on to pediatric obesity lifestyle treatment: a 3-year pragmatic clinical trial. Int J Obes. 2025 Mar;12. 10.1038/s41366-025-01. PMC12095034 40075128

[23] Cole TJ, Lobstein T. Extended international (IOTF) body mass index cut-offs for thinness, overweight and obesity. Pediatr Obes. 2012;7(4):284–94. 10.1111/j.2047-6310.2012.00064.x. 22715120

[24] Reinehr T, Lass N, Toschke C, Rothermel J, Lanzinger S, Holl RW. Which Amount of BMI-SDS Reduction Is Necessary to Improve Cardiovascular Risk Factors in Overweight Children? J Clin Endocrinol Metab. 2016;101(8):3171–9. 10.1210/jc.2016-1885. 27285295

[25] Skelton JA, Beech BM. Attrition in paediatric weight management: a review of the literature and new directions. Obes Rev Off J Int Assoc Study Obes. 2011;12(5):e273–281. 10.1111/j.1467-789X.2010.00803. PMC3079805 20880126

[26] Putri RR, Danielsson P, Ekström N, Ericsson Å, Lindberg L, Marcus C, et al. Effect of Pediatric Obesity Treatment on Long-Term Health. JAMA Pediatr. 2025;179(3):302–9. 10.1001/jamapediatrics.2024.5552. 39836390 PMC11877215

[27] Shai I, Schwarzfuchs D, Henkin Y, Shahar DR, Witkow S, Greenberg I, et al. Weight loss with a low-carbohydrate, Mediterranean, or low-fat diet. N Engl J Med. 2008;359(3):229–41. 10.1056/NEJMoa0708681. 18635428

[28] Rolland-Cachera MF, Thibault H, Souberbielle JC, Soulié D, Carbonel P, Deheeger M, et al. Massive obesity in adolescents: dietary interventions and behaviours associated with weight regain at 2 y follow-up. Int J Obes Relat Metab Disord J Int Assoc Study Obes. 2004;28(4):514–9. 10.1038/sj.ijo.0802605. 14968129

[29] West DS, Gorin AA, Subak LL, Foster G, Bragg C, Hecht J, et al. A motivation-focused weight loss maintenance program is an effective alternative to a skill-based approach. Int J Obes 2005. 2011;35(2):259–69. 10.1038/ijo.2010.138. PMC2974962 20680012

[30] Dhaliwal J, Nosworthy NMI, Holt NL, Zwaigenbaum L, Avis JLS, Rasquinha A, et al. Attrition and the management of pediatric obesity: an integrative review. Child Obes Print. 2014;10(6):461–73. 10.1089/chi.2014.0060. 25496035

[31] Luppino G, Wasniewska M, Casto C, Ferraloro C, Li Pomi A, Pepe G, et al. Treating Children and Adolescents with Obesity: Predictors of Early Dropout in Pediatric Weight-Management Programs. Child Basel Switz. 2024;11(2). 10.3390/children11020205. PMC10887674 38397317

[32] Umano GR, Masino M, Cirillo G, Rondinelli G, Massa F, Mangoni di Santo Stefano GSRC, et al. Effectiveness of Smartphone App for the Treatment of Pediatric Obesity: A Randomized Controlled Trial. Child Basel Switz. 2024;11(10). 10.3390/children11101178. PMC11505602 39457143

