# Supplementary material for: Effectiveness of a digi-physical tool and working method for paediatric obesity treatment in Abu Dhabi: a non-inferiority intervention study using an external historical comparator
Source: BMC Pediatr. 2026 Sep 10;26:868. doi: 10.1186/s12887-026-07623-7 (PMC13573347; doi:10.1186/s12887-026-07623-7)
Supplement: Supplementary file 1 [file 12887_2026_7623_MOESM1_ESM.pdf]

Supplementary material – BMC Pediatrics

Effectiveness of a Digi-Physical Tool and Working Method for Paediatric Obesity Treatment in Abu Dhabi: A Non-Inferiority Intervention Study Using an External Historical Comparator

Louise Lindberg[1,2], Reem Hassan Beck[3], Pernilla Danielsson[1], Timmy Nielsen[2], Asmahan Abdalla[3], Peter Ekstedt[4], Deepti Chaturvedi[3], Nabras Al Qahtani[3], Claude Marcus[1,2], Asma Deeb[3,5]

[1]Division of Pediatrics, Department of Clinical Science, Intervention and Technology, Karolinska Institutet, Stockholm, Sweden
[2]Evira AB, Triewaldsgränd 2, Stockholm, Sweden
[3]Paediatric Endocrine Division, Sheikh Shakhbout Medical City, Abu Dhabi, United Arab Emirates
[4] Swedish Health, Dubai, United Arab Emirates
[5]Faculty of Health & Science, Khalifa University, Abu Dhabi, United Arab Emirates

Corresponding author: Louise Lindberg, louise.lindberg@ki.se

Additional file 1. Baseline characteristics of participants with and without 26-week follow-up data

	**All**			**Abu Dhabi cohort**			**Stockholm cohort**		
	With data<br>n=112	Without data<br>n=55	p	With data<br>n=35	Without data<br>n=25	p	With data<br>n=77	Without data<br>n=30	p
Sex, Females/Males, %	36.6 / 64.4	47.3 / 52.7	0.19	48.6 / 51.4	60.0 /40.0	0.38	31.2 / 68.8	36.7 / 63.3	0.59
Age, mean (min-max)	11.8 (4.1–17.4)	11.9 (5.6–17.4)	0.72	11.4 (6.7–15.2)	11.9 (8.0–15.7)	0.43	11.9 (4.1–17.4)	11.9 (5.6–17.4)	0.99
BMI z-score, mean (min-max)	2.84 (2.19–4.24)	2.90 (2.20–3.77)	0.33	2.96 (2.28–3.75)	2.94 (2.30–3.68)	0.83	2.79 (2.19–4.24)	2.87 (2.20–3.77)	0.30

Differences in proportions were assessed using chi-square tests and differences in continuous variables using t-tests.
